# Editorial: Applications of Next Generation Sequencing (NGS) Technologies to Decipher the Oral Microbiome in Systemic Health and Disease

**DOI:** 10.3389/fcimb.2021.801122

**Published:** 2021-12-22

**Authors:** Thuy Do, Naile Dame-Teixeira, Dongmei Deng

**Affiliations:** ^1^ Division of Oral Biology, School of Dentistry, University of Leeds, Leeds, United Kingdom; ^2^ Department of Dentistry, School of Health Sciences, University of Brasília, Brasilia, Brazil; ^3^ Department of Preventive Dentistry, Academic Centre for Dentistry Amsterdam (ACTA), University of Amsterdam and VU University Amsterdam, Amsterdam, Netherlands

**Keywords:** NGS, oral microbiome, biofilms, dysbiosis, health, systemic diseases

Advances in next generation sequencing (NGS) technologies have revolutionised microbiology in the past few decades enabling researchers to elucidate the composition of the human microbiome. Recently, more effort is being directed at capturing both structural and functional aspects of the microbiome through gathering information on genes, transcripts, proteins and metabolites (Oresic et al., 2004; Muller et al., 2021). Combining several omics approaches with patient information is set to become the norm in future studies and will provide invaluable information to bring our understanding of symbiotic and dysbiotic processes to the next level. In this special issue “Applications of next generation sequencing (NGS) technologies to decipher the oral microbiome in systemic health and disease”, we have compiled 17 papers including mostly original research articles, but also systematic/scoping reviews and a brief research report, that give focus to the oral microbiome and its impact on our systemic health.

The oral cavity harbours a rich and diverse microbial population, which varies greatly within and between individuals. However, it remains relatively stable throughout adulthood (Kilian et al., 2016), despite regular disruptions through diet, oral hygiene, and occasional medication including antibiotics and polypharmacy, if any.

Changes in systemic conditions and associated treatments may influence the oral microbiota, for example hypertension, hyperglycaemia and dysbiosis occurring at other body sites like the gut *via* the oral-gut axis. Several studies in this e-book provide such evidence. Significant alterations in the oral and gut microbial profiles were determined by Shi et al. and were correlated with the severity of rheumatic heart disease. In the study by Kageyama et al., tongue cancer patients undergoing surgical resection were found to have a significantly different salivary microbiota. The mechanisms that underpin the oral-gut microbiome axis and other cross-talks with distant organs such as the lungs, liver, brain, skin and genital tract remain unclear and deserve full investigations (Zhao et al.) (Martinez et al., 2021; Park et al., 2021).

There is also growing evidence that the oral microbiota can influence systemic health (Jia et al., 2018). Specifically, the oral microbiota has been associated with a wide array of conditions such as dementia, depression, obesity, cancer, arthritis, diabetes, gut and skin diseases (Frid et al.) (Maitre et al., 2020; Sedghi et al., 2021; Wingfield et al., 2021). However, from the evidence presented, it is not clear whether changes in microbial profiles associated with oral dysbiosis are a manifestation of disease, or whether they drive the disease process in the oral cavity and elsewhere.

Deeper insights into how the microbiome can influence our wellbeing will help design tailored strategies for disease prevention and treatment. The paper by Zhou et al. is a good example of the use of oral microbiota data to help diagnose carcinomas through a non-invasive and low-cost approach. The acquisition of increasingly large NGS data coupled with biological and medical information is paving the way to the design of more accurate predictive models of health and disease which will enable a comprehensive and personalised medicine approach in our near future. Xie et al. described the use of elastic net models on supra-gingival microbiome data to accurately predict the presence of metabolites. More work is needed to make sense of the complex, multi-dimensional interactions occurring between the host and the microbiome. Computational methods linking microbes with metabolites (either produced or degraded) will be invaluable to learn patterns specific to homeostasis and pathogenesis, with therapeutic implications. Moreover, the ease of access to the oral cavity makes health checks convenient. Attempts at manipulating and controlling the oral microbiota through modulation strategies to reduce local and systemic inflammation, microbial load and maintain oral homeostasis will hopefully lead to similar repercussions systemically and promote overall health.

High throughput sequencing has helped generate large, publicly available datasets of descriptive microbiome profiles which are increasingly combined with functional microbial analyses. Investigations using NGS data combined with proteomics as the one described in Bao et al. are harbinger of future conventional methodologies. Re-analyses and meta-analyses of large datasets are important work which advance the robustness of our analysis methods and our overall knowledge of microbes and their interactions (Cai et al.; de Cena et al.; Jiang et al.; Kang et al.). The inclusion of patients’ clinical data (medical history, systemic health or disease parameters) would also be helpful in deciphering the mechanisms of actions through which the oral microbiome exerts its impact on human health. Most journals impose requirements for NGS studies to provide sequencing data *via* links to repositories. While dealing with manuscripts in this e-book, we have come across two important issues: 1) Incomplete information within presented datasets are common. Kang et al. stated that only 57% of publicly available datasets with accession numbers provided both sequencing and metadata. 2) Clinical data are not always specified in the provided metadata. Within the datasets that provided both sequencing and metadata, we noticed that many manuscripts did not specify patients’ clinical data, for example diagnosis criteria, clinical parameters or medical history. This makes future rigorous re-analyses and meta-analyses difficult for the scientific community, if possible, at all. As we are entering a personalized medicine era, it is crucial that all available data are optimally utilized for comparative analyses of larger and varied patient populations (Garcia et al., 2013; Berg et al., 2020).

Furthermore, taking in account clinical parameters as potential confounding factors for health or disease will be useful in preventing bias when examining omics data. For example, when comparing oral microbial communities in individuals with and without caries, other oral and systemic conditions, e.g. hyposalivation, periodontitis, candidiasis (Lyu et al.) and any sign of inflammation should be taken into consideration (Dame-Teixeira et al., 2021). The study by Bostanci et al. clearly showed how oral dysbiosis fluctuates during the menstrual cycle and detailed the impact of smoking and dietary sugar as risk factors. Inflammatory and immunological impairments also shape the oral microbiome. Lettieri et al. correlated the salivary microbiome with a significant inflammophylic profile in the oral phenotype of syndromic individuals with ineffective cathepsin C and impairment of neutrophils, where traditional periodontal therapy is not efficient.

The field of microbiome research has grown rapidly and has covered many disciplines in the past decade. There is still a lack of consensus in the terminology used to describe some of the methods and microbial communities (Marchesi and Ravel, 2015). We noticed that while most of the papers in our collection used the term ‘16S rRNA gene sequencing’, some refer to the incorrect term ‘16S rDNA sequencing’. Hence, we recommend the standardization of the vocabulary used in microbiome studies (Berg et al., 2020).

In our e-book, we have collected papers on the current trends in oral microbiome research. The studies described integration of meta-omics data with complex biological context of health and disease. Future collaborative efforts are expected to carry out more accurate, rigorous computational methods using increasingly larger, and detailed datasets, which will enable us to predict treatment outcomes and make personalised medicine achievable and accessible.

## Author Contributions

TD, ND-T and DD contributed to the conception of the work, the drafting, writing and critical revision of the article.

## Funding

This work was funded by the UK’s Academy of Medical Sciences Newton International Fellowship (NIF\R5\242).

## Conflict of Interest

The authors declare that the research was conducted in the absence of any commercial or financial relationships that could be construed as a potential conflict of interest.

## Publisher’s Note

All claims expressed in this article are solely those of the authors and do not necessarily represent those of their affiliated organizations, or those of the publisher, the editors and the reviewers. Any product that may be evaluated in this article, or claim that may be made by its manufacturer, is not guaranteed or endorsed by the publisher.
